# Single‐center observation of direct impact of the Tokyo 2020 Games on ambulance emergency response

**DOI:** 10.1002/jgf2.521

**Published:** 2022-01-30

**Authors:** Taku Harada, Dan Watanabe, Haruka Yazawa, Juichi Hiroshige

**Affiliations:** ^1^ 378609 Division of General Medicine Showa University Koto Toyosu Hospital Tokyo Japan; ^2^ Division of Diagnostic and Generalist Medicine Dokkyo Medical University Hospital Tochigi Japan

## Abstract

The burden of the 2020 Tokyo Olympic and Paralympic Games on the local emergency medical system was limited. In addition to the Games were held without spectators, this was due to advance preparation by various organizations and the efforts of local staff, including primary care physicians.

## CONFLICT OF INTEREST

The authors have stated explicitly that there are no conflicts of interest in connection with this article.

To the Editor,

Large‐scale mass gathering events place a heavy burden on the local emergency medical systems.^1^, ^2^ The Academic Consortium on Emergency Medical Services and Disaster Medical Response Plan for the 2020 Tokyo Olympic and Paralympic Games was created in 2016 with the mandate to develop a detailed vigilant plan for emergencies during the games. The planning process included making a comprehensive list of possible medical emergencies, organizing these into meaningful response categories, development of response guidelines for the different categories, presenting the resulting 40 guidelines for acceptance, and, subsequently, preparing information manuals and conducting staff training.^3^

Showa University Koto Toyosu Hospital is an acute care hospital located in the Tokyo Bay area, near the athletes' village and sport venues,^4^ which responds to approximately 4,500 ambulance calls per year. Prior to the event, it was decided that athletes and very important people requiring care would be transported to designated trauma or critical care hospitals, with general conventional staff and spectators diverted to surrounding acute care centers, such as our hospital. To evaluate the direct impact of the Olympic and Paralympic Games on the emergency response in the region for this hospital, we surveyed cases of ambulance transport from the athletes' village and sporting venues over the 30 days of the two events, for the Olympic (July 26 to August 11, 2021) and Paralympic (August 24 to September 5, 2021) Games.

Over this 30‐day period, 412 patients required ambulance transport, including 18 cardiovascular cases, 18 stroke cases, and 38 transfers from other hospitals. A summary of the diagnosis of the 412 cases is shown in Table 1. Of these cases, only 6 were specifically related to the Olympic and 1 to the Paralympic Games of the total of seven cases, with only 2 requiring hospitalization. After the history, only 2 alcohol‐related cases (acute alcohol intoxication and alcoholic ketoacidosis) were specifically related to the games; there were no cases of heat stroke. The diagnoses of the other five cases were dehydration, non‐specific abdominal pain, epilepsy, diverticulitis, and undiagnosed.

**TABLE 1:** Summary of the diagnosis of 412 cases

Summary of the diagnosis of 412 cases	
Minor trauma	43
Febrile seizures	19
Fracture	17
Ischemic stroke	16
Hyperventilation syndrome	14
Acute gastroenteritis	11
Heatstroke	11
COVID‐19	10
Neuro mediated syncope	9
Epilepsy	9
Acute cholangitis and/or cholecystitis	8
Acute coronary syndrome	7
Ureteral calculus	7
Hemorrhagic stroke	7
Pneumonia	7
Atrial fibrillation	6
Psychogenic	6
Constipation	6
Upper gastrointestinal bleeding	5
Acute heart failure	5
Benign paroxysmal positional vertigo	4
Acute alcohol intoxication	4
Dislocation	4
Dehydration	4
Small bowel obstruction	4
Vaccine‐related reaction	4
Gastroesophageal reflux disease	3
Subarachnoid hemorrhage	3
Anaphylaxis	3
Lower gastrointestinal bleeding	3
Acute bronchitis	3
Cervical spondylosis	3
Cervical sprain	3
Cholelithiasis	3
Appendicitis	3
Enteritis	3
Pseudogout	3
Acute pyelonephritis	3
Migraine	3
Hypokalemia	3
Peripheral dizziness	3
Common cold	3
Undiagnosed	39
Miscellaneous	78

The Tokyo 2020 Games were postponed to 2021 owing to the coronavirus disease 2019 pandemic and no spectators were allowed at the events.^5^ As such, the burden on the emergency response system was lower than originally planned for. Regardless, advanced preparation, in combination with the efforts of the local staff for prevention and initial response by primary care physicians and healthcare professionals to minor illnesses limited the impact of the games on the emergency ambulance response needed. We recognize this is only the experience at one acute care center and that the anticipated emergency response burden for the Tokyo 2020 Olympic and Paralympic Games was lowered by the absence of spectators. In these seven cases, there were no environmental injuries such as heat stroke, which could be attributed to the efforts of the local staff, including primary care physicians. Vigilant planning, including appropriate training of local convention staff and primary care physicians on response guidelines, provides an approach for future large‐scale mass gathering events.
